# Combining data to perform population-based observational studies: know your sources. The case of thyroid cancer in Belgium

**DOI:** 10.1186/s13690-022-00803-8

**Published:** 2022-03-10

**Authors:** Brigitte Decallonne, Bérengère Snyers, Nathalie Elaut, Bernard Peene, Julie Verbeeck, Annick Van den Bruel, Harlinde De Schutter

**Affiliations:** 1 Endocrinology department, University Hospitals Leuven, Herestraat 49, 3000 Leuven, Belgium; 2 Belgian Cancer Registry, Brussels, Belgium; 3 EORTC, Brussels, Belgium; 4 Endocrinology department, AZ Sint-Dimpna Hospital, Geel, Belgium; 5 Endocrinology department, AZ Sint-Jan Hospital, Bruges, Belgium

**Keywords:** Administrative data sources, Population-based observational study, Thyroid cancer

## Abstract

**Background:**

Large scale observational studies are crucial to study thyroid cancer incidence and management, known to vary in time and place. Combining cancer registry data with other data sources enables execution of population-based studies, provided data sources are accurate. The objective was to compare thyroid tumour and treatment information between the available data sources in Belgium.

**Methods:**

We performed a retrospective national population-based cohort study. All patients with thyroid cancer diagnosis in Belgium between 2009 and 2011 (*N* = 2659 patients) were retrieved from the Belgian Cancer Registry database, containing standard patient and tumour characteristics. Additionally, information was obtained from the following sources: a) detailed pathology reports b) the health insurance company database for reimbursed performed therapeutic acts (both available for *N* = 2400 patients) c) registration forms for performed and/or planned treatments at the time of the multidisciplinary team meeting (available for *N* = 1819 patients). More precisely, information was retrieved regarding characteristics of the tumour (histologic subtype, tumour size, lymph node status (source a)) and the treatment (thyroid surgery (a,b,c), lymph node dissection (a,b), postoperative administration of radioactive iodine (b,c)).

**Results:**

High concordance in histological cancer subtype (> 90%), tumour size (96.2%) and lymph node involvement (89.2%) categories was found between the cancer registry database and the pathology reports. Tumour subcategories (such as microcarcinoma, tumor ≤1 cm diameter) were more specified in the pathology reports. The therapeutic act of thyroid surgery as mentioned in the pathology reports and health insurance company database was concordant in 92.7%, while reports from multidisciplinary team meetings showed 88.5% of concordance with pathology reports and 86.1% with health insurance data. With regard to postoperative radioiodine administration, reports from multidisciplinary teams and health insurance data were concordant in 76.8%.

**Conclusion:**

Combining registered and/or administrative data results in sufficiently accurate information to perform large scale observational studies on thyroid cancer in Belgium. However, thorough and continuous quality control and insight in strengths and limitations of each cancer data source is crucial.

## Background

Worldwide, thyroid cancer incidence is increasing over time while mortality rates are unchanged or even decreased. This was shown to be, in part, associated to enhanced detection of very small cancers or microcarcinoma (pT1a) [1, 2], and is also the case for Belgium [3]. Additionally, geographical differences have been observed. In a national population-based observational study, we previously showed an association between less conservative treatment of functional and structural thyroid diseases and higher incidence of very low risk cancer, probably as a result of more thyroid surgery [4]. A follow-up study demonstrated an inverse correlation between incidence and the mean weight of thyroid resection specimens across regions, providing additional evidence for a lower threshold for thyroid surgery in regions with high thyroid cancer incidence [5].

**Fig. 1:**
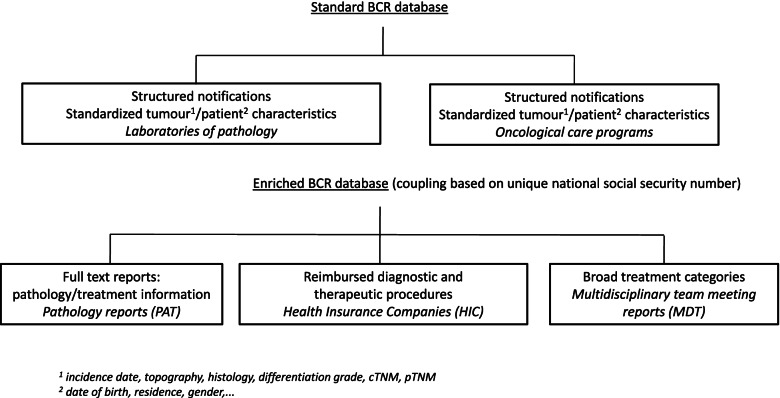
Delivery of cancer data to the Belgian Cancer Registry (BCR)

Population-based observational studies often rely on the combination of data originating from different sources [6–10], underlining the importance of identifying the most suitable source to answer a specific research question. The Belgian Cancer Registry (BCR) is a national population-based registry collecting information on all new cancer diagnoses in Belgium since 2004. The standard BCR cancer registration database relies on an obligatory structured notification for all new cancer diagnoses both from laboratories for pathology and oncological care programs (Fig. 1). These structured notifications are integrated into a broad set of variables including patient and tumour characteristics [11]. In addition to the structured notifications, laboratories for pathology are obliged to provide the BCR with the full text histology or cytology reports (further referred to as PAT) for all malignancies. These reports are useful in quality checks, and can -albeit unstructured- also serve as an additional source of detailed information on a cancer specimen. They are however not systematically consulted during the cleaning and validation process of the BCR standard cancer registration data set. For cases that are discussed in a multidisciplinary team meeting (MDT, i.e. the large majority of new cancer diagnoses), the data provided by the oncological care programs also contain information on given and/or planned treatments that are described in predefined broad categories such as surgery, radiotherapy, chemotherapy, and isotope administration. More detailed treatment information is derived from administrative data of the health insurance companies (further referred to as HIC). HIC data cover charged and reimbursed in- and out-patient medical acts and medication, and can be considered almost complete at the population level since health insurance is mandatory in Belgium. For the coupling of the received information from all data sources, BCR is allowed to use the national social security number as a unique patient identifier [12]. In BCR studies describing real-life cancer care, data originating from PAT, MDT and HIC are regularly used in addition to the standard cancer registration database to describe diagnosis and treatment as detailed as possible. Current practices balance efforts against potential gains, and most often use HIC data given their structured character and availability. HIC data contain more detailed information regarding diagnostic and treatment procedures compared to MDT reports, and on the other hand do not necessitate intensive manual reading as is the case for PAT reports. However, an in depth analysis of the level of agreement between the HIC, MDT, and PAT data has not been done so far.

The objectives of this study were therefore to analyse available national data sources related to thyroid cancer for strengths and limitations regarding information on tumour characteristics (histological subtype, tumour size, lymph node status) and treatment (thyroid surgery, postoperative radioiodine administration) and to investigate the concordance between data sources.

## Materials and methods

### Patients

**Fig. 2:**
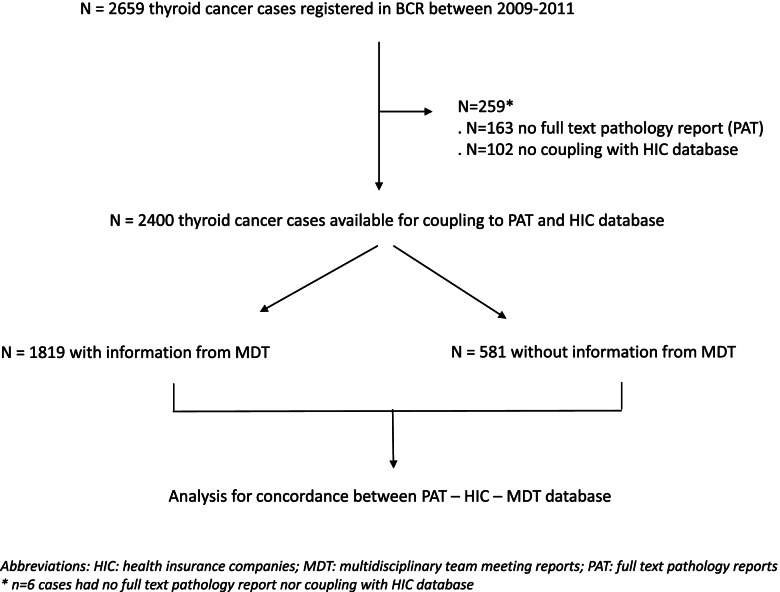
Flow diagram for patient selection

The population used for this study was composed of all thyroid cancers [13] diagnosed in Belgian residents between 1/1/2009 and 31/12/2011 (*N* = 2659). All cases for which a full pathology report was available and which could be coupled with the HIC database were kept for further analyses (*N* = 2400). For 1819 patients, the BCR also received information on given and planned treatments at the time of the MDT meeting (Fig. 2).

The pathology reports that were sent to the BCR by the laboratories of pathology were manually studied on a case-by-case basis. Additionally to the tumour characteristics present in the BCR database, detailed information on extent and type of surgery and lymph node dissection (LND) was extracted (PAT data).

In parallel, HIC data served as a source of information on reimbursed medical acts and medications. Medical acts are registered in the HIC database by nomenclature codes that are publicly available [14]. Dispensed medications are recorded by means of unique national codes which can be converted into WHO-based Anatomic Therapeutic Chemical (ATC) codes. Information on the following acts was derived from HIC: thyroid surgery performed 3 months around incidence date, LND, and postoperative administration of high (30 mCi or more) activity of radioiodine (RAI), as this is only used in selected patients with thyroid cancer and not for treatment of benign thyroid disease.

MDT registrations on given and/or planned treatment categories were also taken into account, specifically for surgery and isotope administration.

For a subgroup of thyroid cancer patients (*N* = 49) with incidence date in 2011 and treated in one academic and one high volume hospital (respectively University Hospitals Leuven and General Hospital Sint-Jan Bruges), all medical charts were manually checked for tumour and patient characteristics and medical acts [15]. This “gold standard” information served as validation material against which the data retrieved from PAT, HIC, and MDT were compared. Coupling between the medical charts information and PAT, HIC and MDT data was established based on the national social security number in line with the legal basis of the BCR [12].

An overview of the different data sources described in this study and the potential information present within each source is given in Table 1.

**Table 1:** Overview of the data sources studied and available type of information

	Histology	pTNM	Surgery	LND	RAI
***PAT***	+	+	+	+	
***HIC***			+	+	+
***MDT***			+		+

*HIC* health insurance companies; *LND* lymph node dissection; *MDT* multidisciplinary team meeting reports; *PAT* full text pathology reports; *pTNM* pathological TNM staging; *RAI* high dose radioiodine

### Statistics

For each studied tumour characteristic or treatment, overall agreement between sources was calculated by concordance as the proportion of cases allocated to the same category in both datasets. In addition, the Cohen’s kappa coefficient (κ-value) was calculated as a measurement of agreement between two databases taking into account the probability of a random agreement [16]. In these calculations, unknown values were not considered. The normal approximation confidence intervals have been calculated based on the asymptotic variance proposed by Fleiss and colleagues [17]. Calculation of estimates and their confidence intervals have been performed by using SAS 9.4 (SAS Institute Inc., Cary, North Carolina, USA). For the interpretation of kappa coefficients, the Landis & Koch classification was applied (< 0: no agreement, 0–0.2: very low agreement, 0.21–0.4: low agreement, 0.41–0.6: moderate agreement, 0.61–0.8: strong agreement, 0.81–1: almost perfect agreement) [18].

## Results

### Thyroid cancer histology, tumour size (pT), and lymph node involvement (pN): concordance between standard BCR database and detailed pathology reports

For the cohort of 2400 patients, 98.1, 92.8 and 95.5% of respectively papillary, follicular, and medullary cancer in pathology reports had the same histology subtype as mentioned in the BCR database. For anaplastic thyroid cancer however, we found only 71.9% of histology confirmation, the remainder of cases being coded as papillary thyroid cancer (10.5%) or as another, less common histology (15.8%) in the BCR database such as sarcomatoid carcinoma, adenocarcinoma, or spindle cell carcinoma.

Comparison of pT categories (pT1, pT2, pT3, and pT4) showed 96.2% concordance between pathology reports and the standard BCR database, with an excellent kappa statistic (0.94, 95% confidence interval (CI) [0.92;0.95]). Pathology reports allowed 410 out of 610 (67.2%) pTx cases in the BCR database to be revised to pT1–4 (Table 2). Likewise, 59.2 and 32.6% of pT1 cases in BCR could be further refined to pT1a and pT1b, respectively, unmasking pT1a as dominant cancer subcategory (results not shown).

**Table 2:** Comparison of information on tumour size (pT) and lymph node involvement (pN) between the Belgian Cancer Registry (BCR) database and detailed pathological reports (PAT)

		***BCR***						
		**pT1**	**pT2**	**pT3**	**pT4**	**pT category reported**	**pTx**	**total**
***PAT***	**pT1**	989	23	2	0	1014	286	1300
	**pT2**	15	315	6	1	337	64	401
	**pT3**	8	3	280	2	293	39	332
	**pT4**	0	1	4	79	84	21	105
	**pT category reported**	1012	342	292	82	1728	410	2138
	**pTx**	31	13	14	4	62	200	262
	**total**	1043	355	306	86	1790	610	2400
		**pN0**	**pN1**	**pN1a**	**pN1b**	**pN category reported**	**pNx**	**total**
	**pN0**	254	3	2	0	259	45	304
	**pN1**	0	33	3	1	37	9	46
	**pN1a**	2	19	75	3	99	13	112
	**pN1b**	3	16	6	115	140	22	162
	**pN category reported**	259	71	86	119	535	89	624
	**pNx**	202	10	3	8	223	1553	1776
	**total**	461	81	89	127	758	1642	2400

Comparison of pN categories showed a concordance of 89.2% with an almost perfect agreement according to kappa statistic (0.84, 95% CI [0.80;0.88]). Furthermore, 19 (26.8%) and 16 (22.6%) out of 71 pN1 cases according to the BCR database could be specified to pN1a and pN1b, respectively, based on detailed information from pathology reports (Table 2).

### Thyroid surgery, lymph node dissection, and radioiodine administration: concordance between detailed pathology reports, reimbursement database, and multidisciplinary team meeting reports

Data on execution of thyroid surgery as mentioned in the PAT and HIC database was concordant in 2224/2400 cases (92.7%), with a moderate kappa coefficient of 0.45 (95%CI [0.38–0.52]) (Table 3A). The vast majority (94.6%) of surgeries mentioned in pathology reports were confirmed by HIC data. This was not the case for 121 remaining cases. For more than half of these (*N* = 68) a second consultation of the HIC data revealed that the surgery took place outside the timeframe of 3 months around the incidence date. Conversely, in 55 cases with thyroid surgery registered in the HIC database, no surgery was reported in the pathology database.

**Table 3:** Comparison of information on execution of thyroid surgery (A), lymph node dissection (B) and administration of radioiodine (C) between administrative data sources

**A.**		***HIC***			**B.**		***HIC***		
		**yes**	**no**	**total**			**yes**	**no**	**total**
***PAT***	**yes**	2140	121	2261	***PAT***	**yes**	414	193	607
	**no**	55	84	139		**no**	116	1677	1793
	**total**	2195	205	2400		**total**	530	1870	2400
		***MDT***			***C.***		***HIC***		
		**yes**	**no**	**total**			**yes**	**no**	**total**
***PAT***	**yes**	1574	173	1747	***MDT***	**yes**	678	215	893
	**no**	37	35	72		**no**	208	718	926
	**total**	1611	208	1819		**total**	886	933	1819
		***HIC***							
		**yes**	**no**	**total**					
***MDT***	**yes**	1516	95	1611					
	**no**	158	50	208					
	**total**	1674	145	1918					

*HIC* health insurance companies; *MDT* multidisciplinary team meeting reports; *PAT* full text pathology reports

Surgery according to HIC corresponds to surgery performed from 3 months before incidence until 3 months after incidence.

Lymph node dissection according to HIC corresponds to all lymph node dissections without timeframe.

Isotope administration according to HIC corresponds to high activity radioiodine (30 mCi or more) administration after thyroidectomy.

Among the 1819 patients with an MDT registration, there was 88.5% concordance for surgery execution when compared with pathological reports but a very low kappa coefficient (0.20, 95%CI [0.14;0.27]). For 51.4% (*N* = 37) of cases where PAT did not mention any surgery, surgery was registered in the MDT database. 70.3% of the latter cases were recorded for surgery in the HIC database. On the contrary, 173 out of 208 patients (83.2%) where no surgery was mentioned in MDT database appeared to have had surgery according to pathology reports, of which 91.4% were confirmed by HIC.

Surgery as mentioned by MDT and HIC was concordant in 86.1% of cases, with a low kappa coefficient of 0.21 (95% CI[0.14;0.27]). For 65.5% (*N* = 95) of patients with no surgery reimbursed by HIC, the MDT database reported a given or planned surgery. Vice versa, 76% of patients for whom the MDT database did not report on given or planned surgery were registered with a reimbursement for surgery in HIC. Among the 253 records showing non-concordance regarding execution of thyroid surgery between the MDT and HIC database, PAT could confirm information of 169 records (66.8%) from the HIC and 84 records (33.2%) from the MDT database.

Information about the performance of LND was 87.1% concordant between PAT and HIC (strong kappa statistic: 0.64, 95% CI [0.61;0.68]). 68.2% of LND mentioned by the PAT were charged to HIC (Table 3B).

Concordance of isotope administration mentioned by the MDT database with therapeutic administration of radioiodine ≥30 mCi according to the HIC database was calculated to be 76.8%, with a moderate kappa statistic of 0.53 (95% CI[0.5;057]) (Table 3C).

### Concordance with information from medical charts

For the 49 thyroid cancer patients for whom the detailed medical chart was available, comparison between pathology reports sent to the BCR and chart information showed 93.6% concordance for pT categories. This percentage fell to 83.0% when taking into account the subcategories pT1a and pT1b. pN categories showed 95.0% concordance.

Concordance for surgery between medical charts, PAT, and HIC data was 100%. Seven out of 49 patients had no given nor planned surgery in the MDT database and 4 had no MDT registration at all. LND as retrieved from medical charts was confirmed for 85.7% of patients according to HIC data (kappa statistics 0.74, 95%CI[0.55;0.94], strong agreement) and for 87.8% of patients according to pathology reports (kappa statistics 0.71 (95%CI[0.52;0.91], strong agreement, Table 4A). Administration of RAI according to medical files was consistent with HIC in 89.8% of cases (kappa statistic of 0.79, 95%CI[0.63;0.96], strong agreement) and with MDT in 82.2% of known cases (kappa statistic of 0.65, 95%CI[0.44;0.85], strong agreement). Among the 30 patients having had RAI according to medical files, 22 had a concordant date of administration in HIC data and 8 had no RAI registration made by MDT (Table 4B).

**Table 4:** Comparison of information on execution of lymph node dissection (A) and administration of radioiodine (B) between medical charts and administrative data sources

**A.**		***Medical chart***			**B.**		***Medical chart***		
		**yes**	**no**	**total**			**yes**	**no**	**total**
***PAT***	**yes**	27	3	30	***HIC***	**yes**	25	0	25
	**no**	3	16	19		**no**	5	19	24
	**total**	30	19	49		**total**	30	19	49
***HIC***	**yes**	24	1	25	***MDT***	**yes**	22	0	22
	**no**	6	18	24		**no**	8	15	23
	**total**	30	19	49		**unknown**	0	4	4
						**total**	30	19	49

*HIC* health insurance companies; *MDT* multidisciplinary team meeting reports; *PAT* full text pathology reports

## Discussion

The current study explored the concordance between available data sources containing tumour and treatment information of thyroid cancers that are used to conduct population-based studies on real-world management of Belgian thyroid cancer patients. In addition, a validation exercise with data directly derived from patient medical charts of 2 hospitals was performed.

In an era in which both health care providers and policy makers increasingly want to assess real-world practices in clinical care, observational studies conducted at the population level gain importance [19, 20]. Such studies are based on data sources containing retrospective and thus readily available information on patient, tumour, and treatment characteristics, preferentially covering a complete population. Although highly informative and representative, these data often lack detailed information or only cover a certain set of characteristics [21]. Furthermore, observational studies frequently rely on administrative data that were a priori not registered for research purposes and hence may suffer from biases (e.g. financial optimization or underreporting bias dependent on level of reimbursement). As a consequence, many studies combine different, preferentially independent, data sources to use information that is as complete and accurate as possible. This may result in overlapping but also inconsistent information, introducing uncertainty on which source is most appropriate.

Comparison of data about histology retrieved by the reading of pathology reports showed more than 90% concordance with the standard BCR database for the most common histological subtypes. In line with the difficulty and inter-observer variability in the diagnosis of follicular thyroid cancer [22], this tumour subtype showed a lower PAT-BCR overlap than papillary and medullary thyroid cancer.

While pT and pN categories generally showed a high concordance between PAT and BCR data, the manual revision of pathological reports resulted in a better specification of pTx and subcategories of pT and pN. On the contrary, pathological reports delivered to BCR tended to less report a pN0 status. This is probably related to the fact that if a LND is performed separately from the thyroid surgery, the report only needs to be provided to the cancer registry in case of malignant lymph nodes.

Concordance on information about execution of thyroid surgery was highest (92.7%) when comparing PAT reports and HIC data. Moreover, the validation exercise with the medical charts revealed that for the two hospitals studied all surgeries were correctly registered in both PAT and HIC. MDT registrations on the other hand showed less concordance with PAT, HIC, and medical charts. This could be due to MDT reports suffering from coding errors or changes in treatment plans.

Regarding execution of LND, concordance between PAT and HIC data could not reach the level as observed for thyroid surgery. This may again be related to the fact that laboratories for pathology are only obliged to deliver reports of malignant samples. The execution of a prophylactic LND in case of a papillary or medullary thyroid cancer [23, 24] with negative lymph nodes will mostly not be present in pathological reports sent to the BCR. Furthermore, differences in interpretation of invoicing rules could explain why some LND mentioned in pathological reports were not found in HIC data, as not all surgeons charge LND and thyroidectomy separately when performed during the same surgery. Comparison with medical chart information showed that for LND both PAT and HIC seem to perform equally in terms of completeness.

We observed differences for tumour and treatment characteristics between the sources of information that were compared, although for most of the variables concordance was between 80 and 90%. Kappa statistics varied more broadly, ranging between 0.20 and 0.94. For most analyses, concordances and kappa statistics scored similarly (e.g. pN categories BCR-PAT: concordance of 89.2%, kappa statistic 0.84). For some however, discrepancies were noted with high concordance rates coinciding with lower kappa statistics. This was especially the case for analyses related to treatments in the complete cohort (Table 3), for example thyroid surgery PAT-HIC with concordance of 92.7% and kappa statistic 0.45, and thyroid surgery MDT-PAT with concordance of 88.5% and kappa statistic 0.20. While these observations seem counter-intuitive, the phenomenon has been explained as a kappa paradox which results from imbalanced marginal distributions of the data [25, 26]. However, as explained by Bexkens et al. [27], this is not a limitation of the kappa coefficient but rather a logical consequence of its purpose to correctly interpret agreement adjusted for agreement by chance alone. We therefore opted to report both concordances and kappa statistics, while at the same time showing all underlying numbers to allow for detailed insights in data.

Each data source has its strengths and weaknesses. The BCR database is composed of a combination of pathological and clinical data, rendering it a rich database. Nevertheless, data available within the BCR database only cover a prespecified set of variables and lack detailed information regarding treatments or subspecifications such as pT1a. Reading of detailed pathology reports clearly adds information on tumour and treatment characteristics but is labour-intensive, hence semi-automatized procedures based on text recognition might facilitate this retrieval in the future. Certain medical acts related to thyroid cancer management such as fine needle aspiration and radioiodine administration benefit less from a manual report exploration, and have HIC data as the most reliable administrative data source.

The current study demonstrated the drawback of restricting the obligation of pathology notifications to the BCR to malignant cases. An extension of deliveries of pathology laboratories to non-malignant cases might result in a better delineation of pN0 cases but also assessment of fine needle aspiration procedures (performance, result, and subsequent management) and therefore be worthwhile to consider. Meanwhile, the BCR recently gained access to hospital discharge data in addition to the data sources already mentioned. In the upcoming time, the potentially added value of this additional data source for the description of diagnostic and therapeutic procedures at the population level will be explored.

Although medical charts are often not available for population-based research, they can serve as the gold standard to validate information derived from registration or administrative data sources. Such a validation phase should cover a representative sample of hospitals and patients, reducing interpretation problems induced by inter-centre coding/reporting/billing practices [28, 29].

## Conclusion

Administrative data sources have the potential to result in accurate information provided thorough and continuous quality control and insight in the strengths and limitations of each cancer data source. This study showed the importance of combining different data sources to ensure the quality of big data used to answer specific research questions in the field of thyroid cancer incidence and large-scale real world management.

## Data Availability

The datasets used and/or analysed during the current study are available from the corresponding author on reasonable request.

## Abbreviations

BCR: Belgian Cancer Registry
HIC: Health insurance company
LND: Lymph node dissection
MDT: Multidisciplinary team meeting
PAT: Full text pathology report
RAI: Radioactive iodine
